# Effect of the down-regulation of the high *Grain Protein Content *(*GPC*) genes on the wheat transcriptome during monocarpic senescence

**DOI:** 10.1186/1471-2164-12-492

**Published:** 2011-10-07

**Authors:** Dario Cantu, Stephen P Pearce, Assaf Distelfeld, Michael W Christiansen, Cristobal Uauy, Eduard Akhunov, Tzion Fahima, Jorge Dubcovsky

**Affiliations:** 1 Department of Plant Sciences, University of California Davis, USA; 2 Faculty of Life Sciences, Plant Sciences, Tel Aviv University, Israel; 3 Aarhus University, Faculty of Agricultural Sciences, Department of Genetics and Biotechnology, Slagelse, Denmark; 4 Department of Crop Genetics, John Innes Centre, Norwich, UK; 5 Department of Plant Pathology, Kansas State University, USA; 6 Department of Evolutionary and Environmental Biology, University of Haifa, Israel

## Abstract

**Background:**

Increasing the nutrient concentration of wheat grains is important to ameliorate nutritional deficiencies in many parts of the world. Proteins and nutrients in the wheat grain are largely derived from the remobilization of degraded leaf molecules during monocarpic senescence. The down-regulation of the NAC transcription factor *Grain Protein Content *(*GPC*) in transgenic wheat plants delays senescence (>3 weeks) and reduces the concentration of protein, Zn and Fe in the grain (>30%), linking senescence and nutrient remobilization.

Based on the early and rapid up-regulation of *GPC *in wheat flag leaves after anthesis, we hypothesized that this transcription factor is an early regulator of monocarpic senescence. To test this hypothesis, we used high-throughput mRNA-seq technologies to characterize the effect of the *GPC *down-regulation on the wheat flag-leaf transcriptome 12 days after anthesis. At this early stage of senescence *GPC *transcript levels are significantly lower in transgenic GPC-RNAi plants than in the wild type, but there are still no visible phenotypic differences between genotypes.

**Results:**

We generated 1.4 million 454 reads from early senescing flag leaves (average ~350 nt) and assembled 1.2 million into 30,497 contigs that were used as a reference to map 145 million Illumina reads from three wild type and four GPC-RNAi plants. Following normalization and statistical testing, we identified a set of 691 genes differentially regulated by *GPC *(431 ≥ 2-fold change). Transcript level ratios between transgenic and wild type plants showed a high correlation (*R *= 0.83) between qRT-PCR and Illumina results, providing independent validation of the mRNA-seq approach. A set of differentially expressed genes were analyzed across an early senescence time-course.

**Conclusions:**

Monocarpic senescence is an active process characterized by large-scale changes in gene expression which begins considerably before the appearance of visual symptoms of senescence. The mRNA-seq approach used here was able to detect small differences in transcript levels during the early stages of senescence. This resulted in an extensive list of *GPC*-regulated genes, which includes transporters, hormone regulated genes, and transcription factors. These *GPC*-regulated genes, particularly those up-regulated during senescence, provide valuable entry points to dissect the early stages of monocarpic senescence and nutrient remobilization in wheat.

## Background

Wheat provides approximately one fifth of the calories in the human diet and is an important source of vegetable protein and nutrients for a large proportion of the world's population. Modern wheat varieties differ in their grain concentrations of N, Zn and Fe [1] and therefore, increases in the nutritional quality of the wheat grain are possible and have the potential to alleviate nutrient deficiencies. In addition, increases in grain protein content (N) are associated with improved pasta and breadmaking quality and, therefore, are rewarded by higher prices in many wheat growing regions.

Wheat grain nutritional content is dependent on the remobilization of amino acids and nutrients from vegetative tissues to the grain during whole plant senescence [2-4]. In monocarpic plants, such as wheat, senescence is a coordinated process acting at the whole-plant level, during which genetically-programmed and developmentally-controlled catabolic activities convert cellular material into exportable nutrients that are remobilized from the leaves to the grain [5,6]. Therefore, nutrient remobilization and senescence are intrinsically interconnected processes, and further improvements in grain nutritional value will require a better understanding of the gene regulatory networks controlling both processes. Unfortunately, this developmental stage has not been studied in great depth, as exemplified by the absence of dedicated senescence libraries in currently available wheat EST resources in NCBI.

Monocarpic senescence is an active process during which the plant must disassemble complex molecules, increase active transport mechanisms and maintain functional conductive tissues, while coordinating the programmed death of depleted leaf cells. Various plant hormones coordinate the initiation and progression of these processes, with abscisic acid (ABA) playing a central role (reviewed in [7,8]). This hormone appears to be the primary signal produced during senescence induction by both drought and high temperature [9]. Several lines of evidence have also indicated important roles for other hormones including jasmonic acid (JA) [10], salicylic acid (SA) [11,12] and ethylene [13].

Leaf cells undergo dramatic physiological and metabolic changes upon the initiation and progression of monocarpic senescence. One of the earliest responses in senescence is the degradation of the photosynthetic machinery. Chloroplasts, which account for approximately three quarters of the organic nitrogen of mesophyll cells, are dismantled early during senescence [6,14]. Proteins, carbohydrates, lipids, and nucleic acids are degraded and catabolic products are supplied to the filling grains [7,14,15]. As RUBISCO and other chloroplast proteins are hydrolyzed by proteolytic enzymes, cellular and phloematic pools of free amino acids increase, accelerating their remobilization to the grains [16]. Remobilization of micronutrients (e.g. Zn, Fe and others) across plant membranes (reviewed in [17]) is mediated by transporters encoded by various gene families. Different transporter gene families have both specific and overlapping abilities to carry different metal cations, potentially acting in concert to regulate the remobilization of micronutrients to the developing grain.

Gene expression studies using microarray technologies in Arabidopsis [11,18,19], *Populus *[20], barley [21] and wheat [22] have shown that senescence is driven by transcription factor networks that regulate the timely expression of hundreds of senescence associated genes (SAGs) illustrating how nutrient salvage requires a complex array of regulatory networks and metabolic pathways [7]. The complexity of the multiple gene networks involved in the initiation and progression of senescence makes it hard to decipher the interactions between individual genes, and the identification of central nodes of the senescence regulatory network.

The existence of a close connection between senescence and nutrient remobilization was also evident in the simultaneous effect of the *GPC-B1 *(*Grain Protein Content 1*) gene on both processes. The 6B chromosome segment including this gene was initially introgressed from wild emmer wheat (*Triticum turgidum *ssp. *dicoccoides*) into durum and common wheat as a source of genetic variation for grain protein content [23], and was later shown to accelerate senescence [24]. Positional cloning of this gene showed that *GPC-B1 *is a NAC transcription factor related to the Arabidopsis *NAC-LIKE, ACTIVATED BY AP3/PI *(*NAP*) gene, which is also involved in the regulation of leaf senescence, as are other members of the NAC family [25-28]. A paralogous gene, designated *GPC-2*, is present on chromosomes 2B (*GPC-B2*) and 2D (*GPC-D2*) of hexaploid wheat and shows a similar transcription profile to *GPC-B1 *[3].

Most commercial pasta and bread wheat varieties have a non-functional copy of *GPC-B1 *and the addition of the functional copy from wild wheat increases N, Fe and Zn concentration in the wheat grain [3]. In contrast, down-regulation of all functional copies of *GPC *in transgenic hexaploid wheat plants expressing a stable RNA interference construct (GPC-RNAi) significantly delayed senescence (> three weeks) and decreased N, Fe and Zn remobilization to the wheat grain (>30%) [3,29].

The *GPC *gene is rapidly up-regulated after anthesis before any visible symptoms of senescence, suggesting that it is an early positive regulator of senescence. Therefore, the available GPC-RNAi transgenic wheat plants and their non-transgenic control represent an excellent entry point to study the gene networks regulating senescence in wheat. Direct cDNA sequencing approaches (mRNA-seq) for transcriptome profiling using Next Generation Sequencing technologies provide high-resolution methods for quantifying gene expression levels on a genome-wide scale [30]. To start deciphering the *GPC-*dependent transcriptome we applied Roche 454 pyrosequencing (454) technology for *de novo *transcriptome assembly and Illumina systems to quantify the expression of 30,497 contigs representing 14,735 wheat genes.

Comparison of the transcriptomes of flag leaves from wild type and transgenic GPC-RNAi lines 12 days after anthesis yielded a set of genes likely regulated by *GPC *during the early stages of wheat monocarpic senescence.

## Results

### *De novo *transcriptome assembly

**Figure 1:**
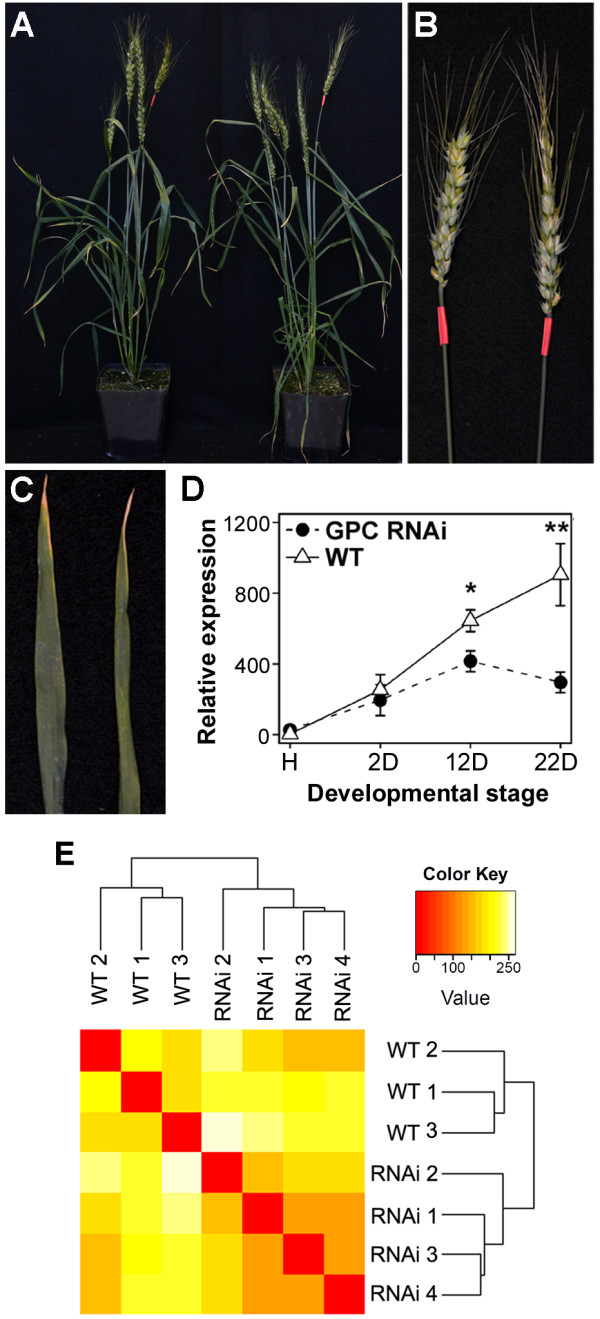
**WT and GPC-RNAi plants 12 days after anthesis**. **(A) **WT (left) and GPC-RNAi plants at 12 DAA used to analyze the *GPC*-dependent transcriptional changes. **(B & C) **Close-up images of the ears (B) and flag leaves (C) from WT (left) and GPC-RNAi plants (right) at 12 DAA. **(D) **Expression profile of the GPC genes relative to *ACTIN *in WT and GPC-RNAi plants across a senescing leaf time course (H = heading, D = days after anthesis). Transcript levels are presented as normalized, linearized values from 10 biological replicates (± SEM) derived from the 2^-ΔΔ*C*t ^method [36], where Ct is the threshold cycle. * P≤0.05, ** P≤0.01. **(E) **Sample clustering based on counts of Illumina reads mapped on 454 contigs. Dendrogram represents the hierarchical clustering of samples as determined by Euclidean distance. The heat map shows a false color representation of the Euclidean distance matrix (from red for zero distance to white for large distance).

The over-expression of the GPC-RNAi construct, under the regulation of the 35S promoter, reduced the transcript levels of all homoeologous copies of *GPC-1 *and its close paralog *GPC-2 *(henceforth, *GPC*) by approximately 35% at 12 days after anthesis (DAA) and by 67% at 22 DAA, relative to the wild type (WT) non-transgenic sister lines (Figure 1D). Senescence in GPC-RNAi plants was delayed by approximately three weeks, as previously reported [3]. To investigate the effect of the down-regulation of the *GPC *transcription factors on the early stages of senescence, we focused our 454 *de novo *transcriptome assembly and Illumina expression studies on RNA samples collected from flag leaves at12 DAA, when *GPC *transcript levels are approximately one third of their level at the transcriptional peak [3]. At this time point, GPC-RNAi transgenic plants showed significantly lower levels of *GPC *transcripts than the WT controls, but neither genotype showed visual symptoms of senescence (e.g. yellowing of the peduncle and a loss of chlorophyll from the leaves, Figure 1A-B, [3]). 'Bobwhite' (the variety used to produce the transgenic plants) frequently shows leaf tip necrosis, but this was observed with equal frequency in both genotypes and was unrelated to the onset of terminal senescence. Even at 22 DAA, when additional samples were collected for qRT-PCR time course studies, there were still no visible signs of senescence in either genotype (Additional file 1 figure S1).

Since no wheat senescence transcriptome was available to use as a reference, we generated a *de novo *transcriptome assembly from flag leaves 12 days after anthesis using the 454 sequencing platform. This technology generates longer transcripts than Illumina, facilitating *de novo *assembly [31]. We sequenced four biological replicates per genotype using two 454 runs (two wild type and two GPC-RNAi transgenic plants per run) and produced 1,469,817 reads, with a median sequence length of 415 nucleotides (Additional file 1 figure S2A). The numbers of reads per plant are described in Additional file 1 table S1).

Eighty percent of the reads (1,179,902) were assembled into 30,696 contigs with a median length of 934 nucleotides (Additional file 1 figure S2B). Contigs were assembled using the default parameters of the GS Assembler (at least 40 bp overlap and at least 90% identical) and, as a consequence, homeologous copies of the same gene were generally assembled within the same contig. Eleven percent of the reads (163,528) were not assembled into contigs and are referred hereafter as singletons (median length 304 nucleotides). Ninety percent of these singletons showed significant similarity to either wheat ESTs (GenBank wheat EST collection: 1,071,199 sequences; BLASTN, E-value ≤ e^-10^) or rice proteins (GenBank rice nr protein collection: 275,532 sequences; BLASTX, E-value ≤ e^-10^). The sequences of the 146,671 singletons that show similarities to either of these databases are available as a multi-fasta file in Supplemental Online Materials (Additional file 2). The residual 9% of the total 454 sequences (126,387) were eliminated based on various quality criteria (see Material and Methods).

Almost 95% of the 454-contigs (29,037) showed high levels of similarity to wheat ESTs (97.4% ± 3.2 average identity ± SD) over most of their lengths (80.4% ± 24.7 average alignment length ± SD). Seventy nine percent of the contigs (24,076) also showed significant similarity (BLASTX, E-value ≤ e^-10^) to sequences in the rice GenBank nr protein collection (79.2% ± 14.8 average identity ± SD) over more than half of their length (63.3% ± 31.4 alignment length ± SD). Among the 912 sequences that did not show similarity to any wheat EST or rice protein sequences, 199 showed high similarity to sequences from insects (172), fungi (14), bacteria (7), and viruses (6) (BLASTN, % identity ≥ 90%; E-value ≤ e^-10^; low complexity filter on). These sequences were considered contaminants and were excluded from further analysis, reducing the number of contigs included in this study to 30,497. Fifty percent of the nucleotides were incorporated into contigs of 1,563 nucleotides or longer (N50 = 1,563). A total of 4,692 contigs (15.3%) were longer than 2,000 nucleotides (Additional file 1 figure S2B). The average coverage was 36.8 ± 1.4 reads per contig ± SD.

The 30,497 contigs were further grouped into 14,735 isogroups. Isogroups are defined in the GS Assembler as a collection of contigs containing reads that imply connections between them, and are expected to include alternative splicing variants as well as homoeologs (A, B and D copies) and close paralogs. The majority of isogroups were comprised of only 1 contig (65.7% 1 contig/isogroup and 18.3% 2 contigs/isogroup). The median length of the sequences forming each isogroup was 685 nucleotides. The identifiers of the assembled sequences, the corresponding isogroups and GenBank accession numbers are listed in Additional file 3.

### Gene expression profiling by mRNA-seq

#### Alignment of Illumina reads to 454 contigs

To determine the differences in transcript levels of the different isogroups between the WT and GPC-RNAi plants at 12 DAA we used the Illumina platform that provided a much greater average sequencing depth per library than the 454 platform (Additional file 1 figure S3). A summary of raw and trimmed Illumina reads is provided in Additional file 1 table S2.

As a quality control, reads were aligned to the 1,138-nt rice waxy-a intron, a sequence present in the pMCG161 RNAi vector but absent in the wheat genome. On average, 858 reads mapped to the waxy-a intron for the four GPC-RNAi samples, with no gaps and no mismatches allowed using Bowtie [32]. As expected, zero reads were mapped to the vector sequence in the sequences obtained from wild type samples WT1, WT2 and WT3. However, 301 reads aligned to the waxy-a intron among the sequences generated from the Illumina library from sample WT4, indicating that the WT4 library was contaminated with RNA from the transgenic GPC-RNAi plants. Therefore, this sample was removed from further analyses.

After trimming low quality regions and vector contaminants and excluding low quality reads (see Methods), a final set of 145 million reads with an average length of 50 nucleotides was obtained from the seven samples. These reads were mapped onto the 30,497 contigs (Additional file 1 table S2) to determine their relative transcript levels (see Materials and Methods). Of the 145 million high-quality Illumina reads, 89 million (61.8%) mapped onto 30,378 contigs (99.6%) corresponding to an average coverage of 22.2× (nt/nt) coverage per library (Additional File 1 Figure S3 and Table S3). Of the 56 million reads (38.2% of the total) which did not align to the 454 contigs, 14 million (9.5% of the total) and 13 million (9.2% of the total) mapped onto 81,230 singletons and 40,996 wheat unigenes (http://www.ncbi.nlm.nih.gov/UniGene/UGOrg.cgi?TAXID=4565), respectively. Taken together, the Illumina reads aligned to contigs, singletons and unigenes, account for 79.9% of the total Illumina reads. The failure to align the 19.5% residual reads to any of the three references sets described above could be explained by (i) the absence of these wheat sequences in all three references, (ii) a higher sequence divergence between reads and reference than the similarity cut-off (maximum of 3 polymorphisms per read used as an alignment parameter), (iii) contamination of transcripts from other organisms, or a combination of these three factors.

This study focuses on the comparison between WT and GPC-RNAi transcript levels at 12 DAA for the 14,735 isogroups assembled with 454-sequencing. Reads mapped to contigs within the same isogroup were summed to obtain the counts per isogroup. Tables including differentially expressed singletons and wheat unigenes are included as supporting online materials for those interested in an expanded dataset of differentially regulated genes (Additional files 4 and 5).

Since the total number of reads obtained for the different biological replicates were not identical, counts were normalized to minimize the effect of this systematic technical variation. We used the normalization procedure implemented in the R/Bioconductor software package DESeq that uses the library median of the ratios between the read count and the geometric mean of each gene as a scaling factor for each library ([33]; Additional file 1 figure S4). All the statistical analyses described below use the normalized data sets.

#### Global characterization of Illumina counts

**Figure 2:**
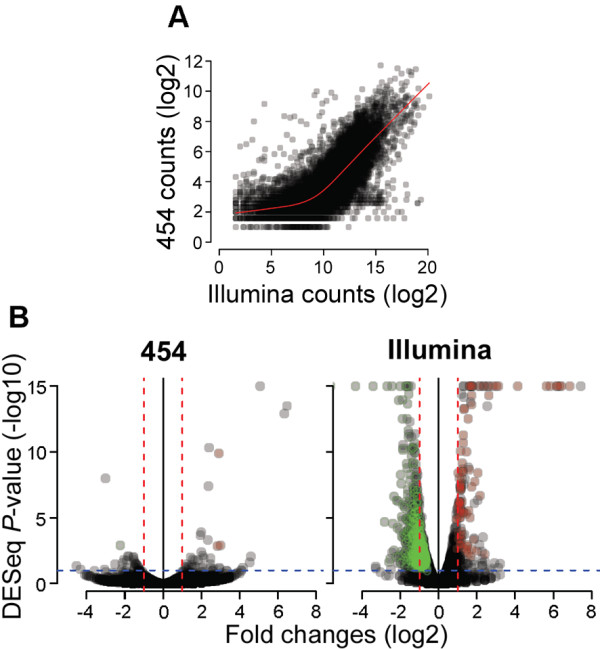
**Comparison between 454 and Illumina mRNA-seq results**. **(A) **Scatter plot of log_2 _transformed 454 and Illumina counts in WT and GPC-RNAi plants. Red lines represent the locally weighted polynomial regression (LOWESS method). **(B) **Volcano plots showing the magnitude of gene expression ratios (log_2_) as a function of the significance of the difference in expression between the two genotypes, is displayed on the *y*-axis [-log_10_(*P*-value at DESeq)]. Vertical red lines delimit two-fold up- and down-regulation and the horizontal blue line corresponds to a *P*-value cutoff of 0.01. Red and green colored circles correspond respectively to up- and down-regulated isogroups significant at both edgeR (*P*≤0.01) and MWW testing (*P *≤ 0.05).

We observed a high Pearson's product moment correlation coefficient between normalized counts of Illumina and 454 sequences (*R *= 0.73, *P *< 0.0001). Figure 2A suggests that this correlation is even better for genes with high Illumina counts. These significant correlations indicate that, overall, similar results were obtained with both technologies. However, the low average counts per isogroup in the 454 data set (5 reads/isogroup) relative to the Illumina data set (863 reads/isogroup) resulted in much higher coefficient of variations in the 454 data set (93%) than in the Illumina data set (20%). Therefore, we used only the Illumina sequencing counts to estimate gene expression levels.

To determine if the down-regulation of the *GPC *gene in the transgenic plants resulted in large changes in the transcriptome, we performed a hierarchical clustering of the seven samples using Euclidean distance as a measure of similarity between expression profiles. As required for distance calculations, normalized values were further transformed to achieve homoscedasticity using the function 'getVarianceStabilizedData' in the DESeq package [33]. Biological replicates were separated into two major clusters, one including the four GPC-RNAi samples and the other including the three wild type plants (Figure 1E). The same grouping was observed when the counts of reads mapping onto singletons and wheat unigenes were analyzed separately indicating a consistent biological signal across these three independent data sets (Additional file 1 figure S5). Principal component analysis (PCA) confirmed the hierarchical clustering results (Additional file 1 figure S6): the primary principal component (PC1) accounted for most of the variation (PC1: 61.6%; PC2: 13.6%; PC3: 7.8%) and clearly separated the replicates from the two different genotypes, which suggests that a large proportion of the variation in this dataset is associated with the differential expression of multiple genes in the two genotypes.

#### Identification of differentially expressed genes

To identify genes differentially expressed between GPC-RNAi and WT plants we used a conservative approach. We first identified isogroups that showed significant differences in normalized counts between treatments in both DESeq [33] and edgeR statistical analyses [34].

**Figure 3:**
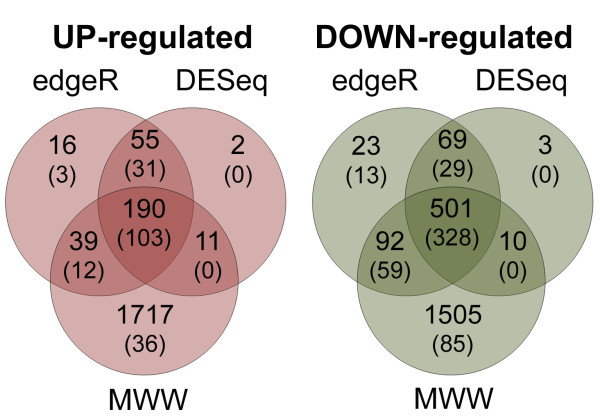
**Comparison of sets of differentially regulated isogroups identified by different statistical testing approaches**. Venn diagrams show the overlapping and unique sets of significantly up-regulated and down-regulated isogroups identify by Mann-Whitney-Wilcoxon (MWW) test or by the R/Bioconductor software packages edgeR and DESeq. Values in parentheses denote isogroups with a ≥ 2 fold differential expression.

The *P*-values generated by both analyses were adjusted for false discovery rates (FDR) across the multiple tests by using the procedure of Benjamini and Hochbergh as implemented in the R/Stats package [35]. Hereafter, the DESeq and edgeR *P *values refer to the adjusted *P *values. Using this criteria, a total of 245 (1.7%) isogroups showed significantly higher transcript counts in the WT relative to the GPC-RNAi samples (up-regulated during senescence) and 570 (3.9%) showed significantly lower transcript counts in the WT relative to the GPC-RNAi samples (down-regulated during senescence) simultaneously in both statistical analyses (*P*≤0.01; Figure 3; Additional file 6). Sixty percent of the 815 differentially regulated isogroups showed more than two-fold differential expression. Gene expression data points in relation to their statistical significance are presented as volcano plots in Figure 2C, which shows an excess of significantly down-regulated genes (green dots) over significantly up-regulated genes (red dots).

To further validate the differences between WT and GPC-RNAi samples, we repeated the same statistical analyses between groups including permutations of the samples. Ten different permutations resulting in two GPC-RNAi and two WT samples in one group and two GPC-RNAi and one WT sample in the other (due to the elimination of sample WT4) were analyzed and averaged. The edgeR and DESeq tests of 10 different permutations showed 22 ± 3 and 18 ± 1 significant isogroups ± SE per permutation (*P≤*0.01), respectively. If we consider only the intersection between the two tests, 15 ± 4 isogroups on average were identified as significantly differentially regulated. These permutation analyses also provided an indirect estimate of the type I error rate in our experimental approach. The 15 isogroups showing statistically significant differences represent 1.8% of the 815 genes that showed significant differences between the GPC-RNAi and WT samples. This proportion of false positives is 80% higher than the theoretical 1% FDR used in our analyses (*P≤*0.01).

The higher than expected rate of Type I error suggested by the permutation analysis prompted us to incorporate an additional statistical filter to reduce the number of potential false positives and narrow down the set of genes for further characterization. We incorporated an additional Mann-Whitney-Wilcoxon (MWW) test between WT and GPC-RNAi (*P*≤0.05), which reduced the number of isogroups with significant differences between treatments from 815 (≥ 2-fold change: 491) to 691 (≥ 2-fold change: 431; Additional file 7). We are conscious that this conservative approach increases the number of real differentially regulated isogroups excluded from further analyses (false negatives), and for this reason we are including both datasets in Additional file 6 (815 genes significantly different in both DESeq and edgeR statistical analyses) and Additional file 7 (691 genes significant in DESeq, edgeR and MWW tests).

### Validation of differences in gene expression by qRT-PCR

qRT-PCR was used to validate the differential expression of 22 isogroups that showed ≥2 fold significant differences in transcript counts between treatments in all three statistical analyses (DEseq≤0.01, edgeR≤0.01 and MWW≤0.05; Additional file 7). From the 431 isogroups fulfilling all four criteria we selected 10 up-regulated and 12 down-regulated genes for further validation. These included isogroups covering a range of different expression ratios between genotypes and genes which were of biological interest to our group, including transporters, signaling components and hormone-related genes (Additional file 1 table S4). We selected *ACTIN *(gene AB19881.1) as an endogenous control gene for gene expression analysis, since this gene has been used successfully in previous studies of senescing leaves [3]. Both Illumina and 454 data confirmed that the expression of *ACTIN*, corresponding to isogroup 01906 in our dataset, is not affected by the expression of the GPC-RNAi construct (fold changes = 1.05, *P *edgeR = 0.86 and *P *DESeq *= *0.90) at this time point. All qRT-PCR data were normalized against the expression of *ACTIN *using the 2^-ΔΔ*C*t ^method [36].

**Figure 4:**
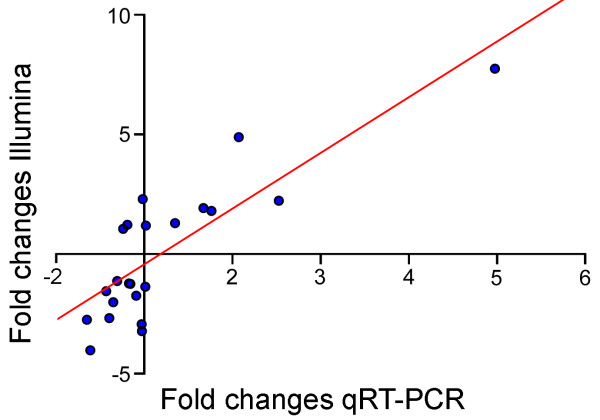
**Correlation between normalized mRNA-seq results and qRT-PCR expression values**. Scatterplot showing ratios of gene expression change from Illumina counts and qRT-PCR. A linear trend line is shown. Values are adjusted so that the origin of the graph is 1 (no change in relative expression).

The transcription ratios between transgenic and wild type samples obtained by qRT-PCR were significantly correlated with the corresponding ratios as determined by normalized Illumina counts (*R*= 0.83, Figure 4). Despite this overall high correlation, only 9 out of the 22 genes tested (41%) showed significant differences (*P*≤0.05) between transgenic and wild type plants when measured by qRT-PCR (Table 1).

**Table 1:** Comparison of the ratios of transcript levels between WT and GPC-RNAi in the Illumina (12 DAA) and qRT-PCR experiments (12 DAA and 22 DAA).

		qRT-PCR ^a^			
		12 DAA		22 DAA	
		
Isogroup	Illumina ratio	Ratio	*P*	ratio	*P*
Isogroup10136	8.745	4.976	0.004	6.472	0
Isogroup08662	5.887	2.11	0.018	2.021	0.001
Isogroup14133	3.303	0.983	0.955	1.208	0.78
Isogroup10811	3.226	2.183	0.034	4.795	0.005
Isogroup12718	2.917	1.67	0.189	1.8	0.018
Isogroup06043	2.797	1.761	0.04	2.088	0.045
Isogroup02905 ^b^	2.292	1.349	0.192	2.8	0.008
Isogroup03083 ^b^	2.218	0.839	0.638	1.904	0.108
Isogroup10053 ^b^	2.19	1.017	0.92	1.094	0.572
Isogroup01211 ^b^	2.052	0.806	0.506	1.582	0.119
Isogroup11278 ^b^	0.471	0.764	0.18	0.726	0.114
Isogroup05843 ^b^	0.448	0.852	0.316	0.827	0.068
Isogroup03470 ^b^	0.445	0.863	0.271	0.845	0.063
Isogroup13287 ^b^	0.422	1.012	0.942	0.853	0.427
Isogroup13088	0.393	0.698	0.050	0.843	0.492
Isogroup06482	0.365	0.916	0.461	0.924	0.605
Isogroup10940	0.333	0.739	0.018	0.631	0.917
Isogroup10620	0.272	0.716	0.029	1.068	0.732
Isogroup13722	0.267	0.606	0.005	0.522	0.336
Isogroup07898	0.254	0.97	0.816	0.882	0.325
Isogroup06574	0.237	0.973	0.782	0.759	0.063
Isogroup07736	0.199	0.62	0.015	0.975	0.912

^a ^Ratios presented as expression in WT/expression GPC-RNAi determined by Illumina counts and qRT-PCR. Ratios higher than one indicate genes up-regulated by senescence and ratios lower than one genes down-regulated by senescence. ^b ^Isogroups with less than 2.5 fold changes.

The high inter-planta variability of the qRT-PCR results may explain, in part, the relatively low rate of validation. The linearized transcript levels of the 22 selected genes showed relatively large coefficients of variation (CV, average 44%) among plants for both genotypes, with significant variation among genes: the 95% confidence interval for CV was between 38% to 50%. Considering these CV values and a Type I error of 5%, the power to detect 2-fold differences between two groups using ten replicates varies from 75% (for a 50% CV) to 95% (for a 38% CV). The low power exhibited by the statistical tests performed for several genes may have contributed to the low proportion of genes that showed significant differences in the qRT-PCR validation analyses. In support of this argument, none of the 8 isogroups with differences between 2 and 2.5 fold were significant in the qRT-PCR analyses (*P*>0.05, Table 1). If we include only the 14 genes with fold changes higher than 2.5 (or lower than 0.4) in the analysis, the rate of validation increases from 41% to 64% (9 out of 14). In addition, two of the thirteen non-significant genes in the qRT-PCR analysis performed on samples collected 12 DAA were significant in a second set of samples collected 22 DAA. The larger relative reduction in *GPC *transcript levels at 22 DAA (67%) compared to 12 DAA (35%, Figure 1D) may explain the increase in significance for some of the selected genes (Table 1).

Taken together, results from our qRT-PCR experiments show a high correlation between qRT-PCR and Illumina results, with a good validation rate among genes that showed differences between the two genotypes larger than 2.5 fold at 12 and 22 DAA.

### Analysis of *GPC*-regulated genes across an early senescing time course

All 11 isogroups which showed significant differences (MWW *P≤*0.05) in transcript levels by qRT-PCR at either 12 DAA or 22 DAA in our initial analyses were selected for an independent, more detailed expression analysis including four time points from heading to 22 DAA.

**Figure 5:**
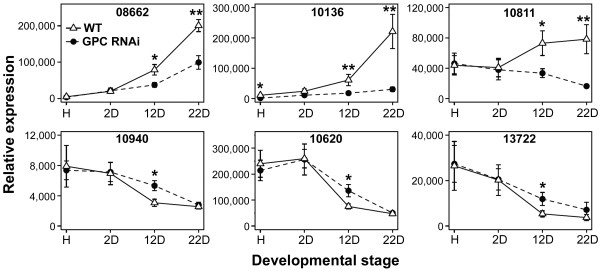
**Transcript levels of selected isogroups across a senescence leaf time course**. Expression levels in WT and GPC-RNAi plants were determined using qRT-PCR at four points across a senescing leaf time course (H = heading, D = days after anthesis). Transcript levels are presented as normalized linearized values using the 2^-ΔΔ*C*t ^method [36], where Ct is the threshold cycle. Values are corrected using the same calibrator across genes and therefore the transcript level scale is comparable between isogroups. Each data point is an average based on ten plants (± SEM). * P≤0.05, ** P≤0.01.

Data from six genes, three up-regulated and three down-regulated are presented in Figure 5. The three up-regulated genes include; isogroup 08662 with homology to a jacalin-like lectin domain-containing gene (ABB51090.1), isogroup 10136 with homology to an ABA induced protein (Q09134.1) and isogroup 10811 with homology to members of the MtN3 Nodulin family (Q0DJY3.2). All three genes showed a gradual increase in the difference in transcript levels between WT and GPC-RNAi during the progression of senescence, paralleling the transcription profiles of the *GPC *gene (Figure 1D). Transcript levels of isogroups 08662 and 10136 are almost undetectable at heading date, whereas isogroup 10811 is highly expressed at heading and only after anthesis displays differential regulation between the two genotypes (Figure 5). The significance of the differences in transcript levels between WT and GPC-RNAi increased between 12 and 22 DAA for all three up-regulated isogroups (Figure 5).

The three down-regulated genes shown in Figure 5 include isogroup 10940 which exhibits high homology to *NAC2*, a rice gene related to the *GPC *gene (BAD09612.1), isogroup 10620 with homology to a chaperone protein (ABF96724), and isogroup 13722 with homology to an LRR disease resistance gene (BAD68095). All three genes show similar expression profiles with an approximate four-fold decrease in expression between heading date and 22 DAA. However, transcript levels in WT plants were reduced to basal levels faster than the GPC-RNAi plants resulting in significantly lower transcript levels in WT than in GPC-RNAi plants at 12 DAA (*P*≤0.05) for all three genes. These differences disappeared at 22 DAA, when both genotypes reached a common low transcript level. The remaining five isogroups showing significant (*P*≤0.05) differential regulation at either 12 DAA or 22 DAA were also tested across a senescing time course in an independent experiment and the data is available as a supplemental online material (Additional file 1 figure S7).

### Gene ontology annotation

To obtain a broad classification of gene functions, GO-slim annotation was assigned based on the GO-slim terms associated with the homologous rice genes [37,38]. Homology searches using BLASTX (E-value < 1*e*^-5^) and the predicted rice peptides as a database showed significant similarities for 78% of the 14,735 wheat isogroups. Among the 815 isogroups significantly up- and down-regulated 12 DAA (*P *≤ 0.01 at both edgeR and DESeq; Table S6), significant matches to known rice proteins were found in a larger proportion among the down-regulated genes (91%) than among the up-regulated genes (40%). Similarly, GO-slim terms for biological process associated with the homologous rice genes (Rice Genome Annotation Project; http://rice.plantbiology.msu.edu/) were assigned to only 10.0% of the 245 up-regulated genes, in contrast to 45.6% of the 570 down-regulated isogroups. Putative rice homologs and assigned GO numbers for molecular functions and biological processes are listed in Additional file 3 and a summary of the relative distribution of the GO-slim categories for the most represented functional terms is presented in Table 2.

**Table 2:** Distribution of the functional grouping of the assembled isogroups based on GO-slim annotation.

Accession	Ontology	Total	**Up-regulated **^a^	**Down-regulated **^a^
GO:0019538	protein metabolic process	5.1	13.9	5.1
GO:0009987	cellular process	7.2	12.4	7.2
GO:0006950	response to stress	13.9	10.2	12
GO:0006810	transport	3.4	8.0	4.3
GO:0009719	response to endogenous stimulus	7.9	8.0	8.5
GO:0009056	catabolic process	2.8	7.3	1.8
GO:0009607	response to biotic stimulus	4.1	5.1	5.5
GO:0007165	signal transduction	8.7	4.4	9.9
GO:0006464	protein modification process	10.6	3.6	8.3
GO:0006350	transcription	2	3.6	1.9
GO:0009628	response to abiotic stimulus cellular component organization	3.2	1.9	2.6
GO:0016043	and biogenesis	3.4	0.7	4.5
GO:0008152	metabolic process	2.8	0.7	1.8
GO:0009058	biosynthetic process	2.7	0.7	3
GO:0006412	translation	3.7	0.0	1.9
	Others	18.5	19.5	21.7

Values are % of the total number of GO-annotated isogroups.

^a^Significantly different between genotypes (*P*≤0.01 edgeR and DEseq).

We observed significant differences in the relative distributions of the functional groups between up- and down-regulated genes (Kolmogorov-Smirnov test: *P *= 0.002) and we also observed a significant change in distribution of functional groups of up-regulated genes compared to the distribution of classes of all annotated isogroups (Kolmogorov-Smirnov test: *P *= 0.0001), which indicates an enrichment of specific functional classes as the transcript levels of the *GPC *gene increase during senescence. In agreement with the accelerated senescence and augmented translocation of minerals observed in the presence of wild type *GPC *expression, we observed an enrichment in the percentages of genes involved in transport (GO:0006810), protein metabolism (GO:0019538) and catabolic processes (GO:0009056), and a reduction in the percentages of genes involved in biosynthetic process (GO:0009058) and cellular component organization and biogenesis (GO:0009628; Table 2) among the up-regulated isogroups.

### Transposable elements

Similarity searches were also carried out against the TREP database to identify transcribed transposable elements (TREP redundant database; http://wheat.pw.usda.gov/ITMI/Repeats/). A total of 748 isogroups (5.1%) and 4,010 singletons (2.5%) showed significant similarity to transposable elements (BLASTN, E-value<*e*^-5^, Additional file 1 table S6). The distribution of different superfamilies of transposable element in our dataset is similar to that observed in the entire wheat EST database and in wheat genomic DNA [39], with Gypsy and Mariner as the most abundant class I and class II elements, respectively. Among the expressed elements, 46 isogroups and 29 singletons were differentially regulated in the two genotypes at both edgeR and DESeq tests (*P *≤ 0.01; Additional file 8). In both datasets, the number of repetitive elements up-regulated during senescence (87% among isogroups and 93% among singletons) largely exceeded the number of repetitive elements down -regulated during senescence (13% among isogroups and 7% among singletons). These proportions were significantly different from a random 50% distribution (X^2 ^tests, *P*<0.0001).

These results support the hypothesis that some transposable elements are activated, potentially through the deactivation of silencing mechanisms [39] during senescence as observed under conditions of biotic and abiotic stresses [40-42]. Differences were detected in the distribution of different classes of retroelements between the complete data set and the subset of elements up-regulated during senescence, both for the isogroup (Kolmogorov-Smirnov test: *P *= 0.06) and the singleton set (Kolmogorov-Smirnov test: *P *= 0.02). The up-regulated repetitive elements displayed enrichment of Gypsy elements in the isogroup set and of Mariner elements in the singleton set.

## Discussion

Monocarpic senescence is characterized by a developmentally regulated set of physiological changes encompassing extensive alterations in gene expression [21,22]. These changes are intrinsically linked with the remobilization of nutrients and carbohydrates from the leaves to the grains, as shown by the simultaneous alteration of senescence and remobilization in isogenic lines with different dosages of active *GPC *genes or in transgenic RNAi plants with different *GPC *transcript levels [3].

### Global analysis of gene expression during early senescence

Based on the early up-regulation of *GPC *after anthesis and the delayed senescence of the GPC-RNAi plants [3], we hypothesized that this transcription factor is an early regulator of monocarpic senescence. To test this hypothesis, we selected an early time point after anthesis when there are no visible phenotypic differences between WT and GPC-RNAi plants (Figure 1A-C), but when the *GPC *transcript levels are significantly different between WT and GPC-RNAi (Figure 1D). The 35% reduction in *GPC *transcript levels observed 12 days after anthesis in the transgenic plants relative to the WT (Figure 1D) was associated with large-scale differential gene expression affecting roughly 5.5% of the isogroups included in this study (using a stringent criteria). Most of these differences disappear when the same analyses were made between reshuffled groups including mixtures of wild type and GPC-RNAi samples, confirming the existence of real biological differences. The fact that the differences in *GPC *transcript levels affect a large number of genes was also reflected in the cluster and principal component analyses of isogroups (Figure 1E), singletons and unigenes (Figures S5 and S6). In all the analyses the transcriptomes were divided into two major groups defined by the *GPC *genotype.

Since the down-regulation of the *GPC *genes has multiple pleiotropic effects on senescence and nutrient remobilization [3] and affects the overall timing of the senescence process, it is possible that many of the genes differentially regulated in the transgenic plants would also be differentially regulated between different time points of the senescence process in wild type plants. However, we currently do not know if the genes affected by the *RNAi *down-regulation of the *GPC *genes include the complete set of genes differentially regulated during senescence or just a subset of them. This will depend on how early the *GPC *gene is located within the hierarchy of transcription factors that coordinate the senescence response and on the presence of feedback regulatory loops initiated downstream of *GPC *and that activate earlier regulatory steps.

Expression analysis of six selected candidates across an early senescing time course (Figure 5) suggests that the selected time point for the mRNAseq analysis (12 days after anthesis) provided a good snapshot of the transcriptional changes associated with the down-regulation of the *GPC *gene. At 2 DAA, none of the six genes analyzed by qRT-PCR in Figure 5 showed significant differences in transcript levels. Conversely, transcript level differences at 22 DAA were larger in all three up-regulated genes, but not significant for all three down-regulated genes since their expression levels in both WT and GPC-RNAi samples had been reduced to similar lower levels compared to heading date by this time point (Figure 5). Among the three additional down-regulated genes presented in Additional file 1 figure S7, isogroup 06043 also showed larger differences at 12 DAA compared with 22 DAA, but both differences were significant. Thus, an analysis limited to the 22 DAA might have resulted in an underestimation of the number of down-regulated genes. In addition, as senescence progresses, the initial regulatory effects of the *GPC *gene are likely to be expanded by other transcription factors which are induced at this and later time points, thus complicating our analysis.

Previous microarray-based approaches have provided an initial picture of the transcriptome changes during senescence. However, most of these studies were carried out in dicot species [11,18-21,43,44], and only a few such studies have been performed in temperate cereals [21,22]. These studies resulted in the identification of hundreds of genes which are activated as senescence progresses, including those with putative roles in metabolism and translocation [38]. However, the use of microarrays can be limiting, particularly when analyzing a relatively sparsely studied stage of development such as senescence.

### *De novo *transcriptome assembly

Unlike microarrays, which are limited to the genes printed on the chips, mRNA-seq represents an open platform that is capable of detecting novel transcripts, provided that they are expressed at levels compatible with the sequencing depth. This advantage of the mRNA-seq approach is particularly valuable considering the limited current sequence information of wheat genes expressed during senescence and the lack of an assembled wheat genome. Of the 1,071,335 wheat ESTs present in GenBank at the time of this analysis, none are derived from flag leaves collected after anthesis and there are only two annotated wheat SAGs present in the Leaf Senescence Database, one being the *GPC *gene itself [45].

About 5% of the genes assembled from the 454 sequence (excluding the singletons) (~1,460 genes) were not found in the NCBI wheat EST collection (BLASTN, E ≤ e^-10^), suggesting that the effort to generate a *de novo *transcriptome assembly provided novel information. It is possible that a larger proportion of novel genes can be identified from later stages of the senescence process. The limited genomic information available for senesce genes is also reflected by the small proportion of up-regulated transcripts (10%; edgeR and DESeq *P *≤ 0.01) that we were able to annotate using GO Slim (based on putative rice homologs) when compared to the proportion of the annotated down-regulated transcripts (46%; edgeR and DESeq *P *≤ 0.01).

A high proportion of reads from the 454 sequencing runs (82.9%) were assembled into contigs with a high N50 and an average contig length of 1,216 bp, which is longer than those reported before, which range from 197 bp [46] to 500 bp [47]. The accuracy of the sequencing and assembly was confirmed by BLAST similarity searches, both in terms of identity and alignment length. The assembled transcripts are publically available through the TSA division of GenBank, and the singletons as a multi-fasta file in the SOM (since only assembled contigs are accepted in the TSA division).

The assembly of the transcriptome of a polyploid species such as wheat poses additional problems that are not encountered in diploid species. Homeologous transcripts of the three wheat genomes are approximately 97% identical [48] and, under the assembly parameters used in this study, are usually merged into chimeric contigs. Therefore, when mapping reads to the contigs it is important to adjust the number of mismatches to tolerate the average differences generated by genome divergences. In our case, we mapped reads with up to 3 nucleotide differences in 50 bp average reads, which will result in the inclusion of reads with more than 94% identity. Some of the unmapped reads may represent reads that are more divergent than the genome present in the isogroup used as reference. The objective of this study was the identification of differentially regulated genes, and therefore the collapse of the three wheat homeologs into single contigs was a conscious decision. Future studies aiming to characterize transcript levels of specific homoeologs will require a much higher coverage and higher stringency to assemble contigs for the different homoeologous groups.

Based on the observed Ct values from qRT-PCR experiments, the assembled transcriptome includes genes with transcript levels as low as 1/36 of *ACTIN *transcript levels (e.g. isogroup 12718). Even though we obtained 12-16 million reads per sample, ~38% of the Illumina reads were not mapped to the 14,735 isogroups. Approximately half of those unmapped reads were subsequently mapped to the singleton set (9.5%) or to the wheat GenBank UniGene set (9.2%, among those not mapped to contigs or singletons), confirming that our 454 contigs represent a transcriptome limited to genes expressed at relatively high levels. The average number of counts mapped onto the singletons was one order of magnitude lower than those mapped onto the assembled contigs (Additional file 1 figure S3). This result suggests that, on average, the transcript levels of the genes included in the singleton dataset are lower than those included in the assembled contigs. The residual unmapped reads may correspond to genes missing from all three reference wheat datasets, that have more than three polymorphisms with the reference sequence (cutoff limit), or that correspond to foreign DNA contamination and/or sequencing errors [49].

A BLASTX comparison of our dataset of 815 differentially regulated genes with the Leaf Senescence Database [45] showed significant similarities (E-value<1*e*^-5^) for 181 genes (24 up-regulated and 157 down-regulated). This result suggests that the differentially expressed genes present in our dataset include both a core of known SAGs as well as a large set of previously uncharacterized SAGs. However, one additional reason why similarities might not have been detected is that almost 90% of the sequences deposited in the leaf senescence database are from dicotyledonous species. Only 9% of the sequences are from rice and 1% from other grasses [46], http://www.eplantsenescence.org/ as of April 20^th ^2011). Therefore, our dataset expands considerably the number of genes with potential roles in regulating senescence in the monocots. We hope that these datasets will become useful tools for research projects investigating monocarpic senescence of cereals.

### Gene expression profiling by sequencing

A comparison of the number of Illumina reads mapping to a particular gene in wild type and GPC-RNAi transgenic samples is a good indicator of its relative transcript levels [50]. The validity of this approach was confirmed by a high correlations between normalized Illumina counts and both 454 and (Figure 2) qRT-PCR data (Figure 4), and by the separation of the transcriptomes of GPC-RNAi and WT plants into the same two different clusters when contigs, singletons and unigenes were analyzed separately (Figures 1E, S5 and S6). From our results it is evident that expression level and sequencing coverage are both important determinants of the precision of the gene expression measurement since the variation in read counts between libraries is reduced as read count increases (Figure 2B). 454 data displayed a low coverage, with small counts particularly for genes with low transcript levels and a large variation between replicates. In contrast, the precision provided by the high coverage of Illumina sequencing resulted in sufficient sensitivity to detect significant differences (*P *≤ 0.01 at both edgeR and DESeq tests) for changes in gene expression as low as 50%. Genes with low expression levels are generally difficult to measure accurately, resulting in low validation rates across platforms [51,52].

The Illumina and qRT-PCR platforms have different strengths and weaknesses. qRT-PCR provides the sensitivity to detect genes at very low expression levels, which would require extremely deep Illumina sequencing, but has limited power to detect differences lower than 2-fold, unless a large number of biological replicates are used (>10 for some of the coefficients of variation observed in our study). In contrast, Illumina sequencing has the power to detect small differences in expression but lacks the sensitivity to detect genes with very low transcript levels (unless a very deep coverage is used). The relatively low power of qRT-PCR to detect differences close to 2-fold (only 41% validated genes) is reflected in the improved proportion of significant differences in the qRT-PCR test observed when the comparisons were limited to genes with more than 2.5 fold differences in transcript levels (64% validated genes). However, the high correlation between qRT-PCR and Illumina transcript level ratios between genotypes (*R *= 0.83, Figure 4) suggests that there are significant similarities between the two datasets.

The time and cost of validating genes individually by qRT-PCR, together with its relatively low power compared with new mRNA-seq technologies, will probably result in the gradual replacement of qRT-PCR as the golden standard for validation of global expression studies. We envision next-generation mRNA-sequencing analyses of transcriptomes in independent experiments using different genetic stocks as a more efficient strategy to validate hundreds of differentially regulated genes simultaneously. As next generation sequencing approaches become increasingly affordable and technologies and data analysis tools mature, replicated mRNA-seq experiments will likely become a common validation strategy.

### Biological significance of *GPC*-regulated genes

The list of putative *GPC*-regulated genes generated in this study provides a valuable entry point to dissect the pathways regulating senescence and nutrient translocation in wheat. However, the lack of functional information for a large fraction of the differentially regulated transcripts limited our ability to perform a more comprehensive bioinformatics analysis of the biochemical and signaling pathways differentially regulated during senescence. Because of this limitation, the following discussion focuses on the variation in the relative proportions of several functional categories, and on a more detailed study of several genes that represent examples of up-regulated and down-regulated genes within different functional categories.

Down-regulated isogroups comprise 70% of all differentially regulated genes (Figure 3) and are likely to include genes coding for processes no longer required during this final stage of the plant's development. Within our dataset, this is evident in the large-scale down-regulation of signaling components (Table 2), including wall-associated kinases (WAKs) of which several members are down-regulated (directly or indirectly) by GPC (E.g. isogroups 14599 and 13361, (DEseq *P*≤0.01, Edge R *P*≤0.01 and MWW *P*≤0.05, Additional file 3). In addition, GPC may play an active role in shutting down photosynthetic processes during the early stages of senescence as suggested by the down-regulation of several genes encoding components of the photosynthetic machinery in the WT samples (higher GPC levels) relative to the GPC-RNAi. The down-regulated genes include those encoding the small subunit of Rubisco (isogroup 01798, MWW *P*≤0.05, Additional file 3) and at least eight putative genes encoding chlorophyll a/b binding (CAB protein), a well-characterized gene closely associated with the onset of senescence (e.g. isogroups 11346, 11739 and 11151(DEseq *P*≤0.01, Edge R *P*≤0.01 and MWW *P*≤0.05) Additional file 3; [53,54]). The finding that a NAC transcription factor is also significantly down-regulated as *GPC *expression increases (isogroup 10940, Figure 5) shows that the differential transcriptional regulation of different members of the NAC family may play diverse roles as the plant transitions into senescence.

In contrast, genes which are up-regulated during senescence are likely to include those playing an active role in driving the onset of senescence and activating the transport networks required for the remobilization of nutrients to the grain and are thus of greater biological interest to enhance grain nutritional value. Among the genes most highly up-regulated in this study is a gene encoding a JA-induced jacalin-like lectin domain protein (isogroup 08662, Figure 5). Transcript levels of this gene are almost undetectable at 2 DAA and it is only as *GPC *expression increases, that its expression is induced. Differences in this gene's transcript levels between WT and GPC-RNAi plants increased from 12 to 22 DAA (Figure 5) paralleling the results observed for the *GPC *transcripts (Figure 1D). Jasmonic acid has been shown to induce leaf senescence in Arabidopsis [10] and several JA biosynthetic genes are up-regulated during senescence [53]. JA-responsive SAGs have been identified in independent microarray studies [55-57] including several genes in the jacalin lectin gene family. The strong up-regulation of this gene by GPC in addition to a significantly up-regulated JA-inducible protein (isogroup 09977, edgeR and DESeq *P *≤ 0.01, MWW <0.05, Additional file 3) suggests that JA may play an important role in the onset of senescence in wheat.

The hormone ABA also plays a well-documented role in the induction of senescence in other plant species [8,13]. Our finding that a putative ABA inducible protein is among the most highly up-regulated genes (isogroup 10136, Figure 5) shows that genes induced by this hormone may also be involved in the induction and progression of senescence in wheat. As with other up-regulated genes, transcript levels increase at 12 DAA and onwards in accordance with increasing *GPC *expression. A closer functional study of these wheat genes has the potential to provide new insights into the roles played by JA and ABA during monocarpic senescence and on their interactions with the *GPC *genes.

The increase in *GPC *expression is also associated with an increase in the translocation rate and more efficient remobilization of several important micronutrients to the developing grain [3,29]. Since a large proportion of the nutrients found in the grain arise from the dismantling of leaf-cell components during senescence [58], one possible explanation for the improved remobilization is a more rapid and complete breakdown of complex leaf molecules resulting in an overall increase in the concentration of simpler molecules ready to be transported. In agreement with this, our gene ontology annotation revealed an enrichment of genes involved in protein metabolism and in catalytic processes among those significantly up-regulated (Table 2).

Studies using steam girdling to induce senescence in barley leaves have indicated a prominent role for proteases in regulating this cellular breakdown, particularly those of the cysteine protease class [59,60]. A TBLASTN search (E-value ≤ *e*^-10^) using all characterised barley proteases against our isogroup, singleton and unigene sets yielded 150, 107 and 149 wheat proteases, respectively (data not shown). Among these 406 proteases only seven with homologs in the unigene assembly (CK209601, CK194339, CA646317, CK218017, CK214606, CK205578, CK167111; Additional file 5) showed significant up-regulation in the WT relative to the GPC-RNAi samples (DEseq *P*≤0.01, Edge R *P*≤0.01 and MWW *P*≤0.05). One possible explanation for the relatively limited up-regulation of proteases in our dataset could be the differences in experimental approaches (naturally-induced monocarpic senescence vs. steam girdling induced senescence). Alternatively, it is possible that *GPC *plays a limited role in the up-regulation of proteases and that, at 12 DAA, the genes coding for other proteases are not yet differentially regulated. Since visible signs of senescence in the leaves are still absent even at 22 DAA (Additional file 1 figure S1), the cellular degradation and metabolism mediated by proteases may simply be occurring at a later stage of senescence and thus beyond our detection.

An obvious way to increase nutrient remobilization is to increase the expression or activity of transporter genes responsible for the transport of nutrients to the grain. For example, over-expression of a gene encoding a member of the ZIP transporters in barley resulted in increased levels of zinc in the developing grain [61]. Within our transcriptome data, there are a greater proportion of genes with transporter activity among those up-regulated by GPC than among those down-regulated by GPC or not affected by GPC (Table 2). The up-regulated transporters include one member of the NRAMP family (isogroup 01654, MWW *P*≤0.05, Additional file 3) and one from the ZIP gene family (isogroup 02825, MWW *P*≤0.05, Additional file 3). NRAMP proteins have been shown in plants to act as multiple divalent metal cation transporters, accepting ions including iron [62], aluminium [63] and cadmium [64]. In plants, the ZIP family of transporters have been well-characterised as metal ion transporters and several members have been shown to be up-regulated during senescence [53].

Based upon rice annotation, several genes of the nodulin family are significantly up-regulated during wheat senescence. The most highly up-regulated member of this family is from the MtN3 nodulin class which is significantly up-regulated in the WT relative to the GPC-RNAi at 12 DAA, and which increases further at 22 DAA (isogroup 10811, Figure 5). A close homolog in rice has been shown to interact with two Cu transporters (COPT) [65,66] suggesting the possibility that the homologous genes identified in our screen may play a similar role in the remobilization of nutrients during senescence. Additional experimentation will be necessary to elucidate the role of wheat nodulins during monocarpic senescence.

While the approach described in this study provides a starting list of differentially expressed genes to dissect the early stages of GPC-regulated senescence, we are currently unable to distinguish between those genes directly regulated by GPC and those which are regulated indirectly through intermediate genes. Chromatin immunoprecipitation experiments complemented with *in vitro *identification of the GPC specific DNA-binding sequences will be necessary to address this question [67,68].

## Conclusions

The comprehensive overview provided by mRNA-seq is well-suited to explore the complexity of the regulatory networks and metabolic and physiological events associated with monocarpic wheat senescence and nutrient remobilization. This study confirms the hypothesis that the *GPC *gene is an early regulator of a complex regulatory network that includes hundreds of genes affecting both monocarpic senescence and nutrient remobilization from the leaves to the grain. In addition, our study shows that the onset of monocarpic senescence is characterized by the down-regulation of a large number of genes, which are likely to be no longer necessary. Although genes up-regulated during senescence represent a small proportion of the differentially regulated genes identified in this study, they will likely provide the most interesting targets to identify the active processes triggered during monocarpic senescence. These active processes are most likely required to guarantee an orderly disassembly of the contents of the leaf cell and the subsequent efficient remobilization of these nutrients to the developing grains.

The early time point of the senescence process selected in this study (12 DAA) proved to be very informative, but a more detailed study of the senescence time course will be required to better understand this dynamic process. In addition, the GPC-RNAi transgenic plants showed a simultaneous down-regulation of the *GPC-1 *and *GPC-2 *genes making it impossible to differentiate the individual contributions of these paralogous genes. We have developed a set of tetraploid wheat *GPC *mutants (*gpc-1*, *gpc-2 *and double *gpc-1/gpc-2*) using TILLING (Targeting Induced Local Lesions in Genomes [69]) with which we plan to carry out a more extensive characterization of the senescence time course using mRNA sequencing. The same tetraploid TILLING population [69] can also be used to generate loss-of-function mutants for selected GPC up-regulated genes to determine their functions and to initiate a systematic dissection of the regulatory network controlling senescence in wheat. We have initiated the TILLING of the JA-induced jacalin-like lectin domain protein as an initial step in the dissection of this complex regulatory network.

## Methods

### Plant material and RNA extraction

Transgenic plants for the GPC-RNAi construct (*T. aestivum *cv. Bobwhite) and the wild-type controls [3] were grown under long-days (16 h light 8 h dark). Single entire flag leaves were sampled 12 days after anthesis (12 DAA) and immediately frozen in liquid nitrogen. The same plant material was used for library construction and qRT-PCR validation, using four independent biological replicates for library construction and a total of ten biological replicates for the qRT-PCR validation experiments. The leaves were ground and RNA samples were extracted using the RNeasy Plant Mini Kit (QIAGEN). Concentration and purity of total RNA was checked on a Nanodrop Spectrophotometer. RNA integrity was evaluated by standard formaldehyde agarose gel electrophoresis.

### 454 mRNA sequencing

The sequencing of full-length cDNA libraries was performed using a GS FLX sequencer (Roche) in Kansas State University Integrated Genomics Facility following the standard single read shotgun 454 sequencing protocol with Titanium chemistry (Roche). First-strand cDNA synthesis was performed according to SMART cDNA synthesis technology (Clontech Laboratories, Inc.) using modified 3' SMART CDS Primer II A (5'-AAGCAGTGGTATCAACGCAGAGTACTTTTGT(9)C T(10)VN-3') and SuperScript III reverse transcriptase (Invitrogen). Double-stranded cDNA was amplified by long-distance (LD) PCR using Advantage 2 PCR Enzyme System (Clontech Laboratories, Inc). Amplification was performed on a thermal cycler (Applied Biosystem) with the following PCR parameters: 95°C - 1 min. followed by 16 cycles of 95°C - 15 sec., 65°C - 30 sec., 68°C - 6 min. The quality of double-stranded cDNA was checked by running on a 1.1% agarose/EtBr gel in 1X TAE buffer and was purified using QIAquick PCR Purification Kit (QIAGEN).

cDNA sequence assembly and trimming of adaptors used for library construction were performed using GS Assembler (Roche) with default parameters for overlap detection. These parameters include a minimum overlap of 40 bp and a minimum overlap identity of 90%. Since the wheat A, B and D genome coding regions are approximately 97% identical [48], the 454 contigs presented here are likely to include the combinations of the different homoeologs as well as intra-genomic paralogs. Reads identified as outliers by the GS De Novo Assembler and reads shorter than 50 nt after trimming were excluded from the assembly process.

The number of 454 reads within each isogroup was counted using a custom PERL script using the data in ACE file generated by the GS De Novo Assembler. Assemblies were deposited in GenBank (TSA division) under accessions HP608076 - HP639668 (TSA project 59945; Additional file 2). BLAST analyses were run locally using BLAST 2.2.21 (NCBI).

### Illumina mRNA sequencing

Amplified cDNA synthesis was performed according to the mRNA sequencing protocol from Illumina (Part # 1004898 Rev. D) and using 10 μg total RNA as starting material. The initial targeted insert size ranged between 150 to 250 bp, but shorter fragments were incorporated during the purification process as reflected in the subsequence analyses. Sequencing was carried out on an Illumina Genome Analyzer II at the DNA Technologies Service core at UC Davis (http://genomecenter.ucdavis.edu). One paired-end sequencing runs were carried out using 85 cycles. The primary output of the Illumina pipeline (qseq files) was used to extract high quality sequences. The parser, http://code.google.com/p/atgc-illumina/wiki/Illumina_QSEQ_Parser analyzed quality scores in qseq files and trimmed everything after the first failed score, 'B' (see http://code.google.com/p/atgc-illumina/wiki/Illumina_Quality_Scores). Upon trimming, we filtered out sequences shorter than 40 nt and those whose GC content was not within the 20% - 80% range. FASTA files with high-quality trimmed sequences were used for downstream analysis. Low quality sequences and vector/adaptor contaminants were removed using custom scripts available from http://code.google.com/p/atgc-illumina/. The Illumina reads were deposited in the National Center for Biotechnology Information's Gene Expression Omnibus (GEO) and are accessible through GEO (accession n. GSM632785-91; http://www.ncbi.nlm.nih.gov/geo/query/acc.cgi?acc=GSE25759).

High quality reads were mapped to the 454 assemblies, singletons and unigenes (http://www.ncbi.nlm.nih.gov/UniGene/UGOrg.cgi?TAXID=4565) using Bowtie V0.12.5 [32] global alignment mode and allowing a maximum of three mismatches (parameters: -k 1 --best -v 3 -f -- sam--sam-nohead). Results were confirmed with SOAPaligner/SOAP2 [70]. SAM output files were parsed with custom Perl scripts to count the number of reads mapping to a single contig/isotig. Reads matching isotigs and contigs within isogroups were summed to obtain the counts per isogroups. Isogroup normalized counts from wild type and GPC-RNAi transgenic plants were compared using both DESeq [33] and edgeR statistical analyses [34]. Both programs assume a negative binomial distribution for the count data, but differ in their models for estimating the distribution parameters from the data. In particular, edgeR uses a single-value dispersion estimate of the variance, whereas DESeq estimates the variance locally, using different coefficients of variation for different expression levels assuming that genes with a similar expression level also have similar variance across replicates (for details see [33] and [34]).

### qRT-PCR

The sequence from each target isogroup was used to screen the available wheat NCBI EST database (http://www.ncbi.nlm.nih.gov/). Primers were designed using Primer3 software (http://frodo.wi.mit.edu/primer3/) based on the isogroup sequence, or if available, on the consensus sequences from ESTs or contigs from the different wheat genomes. Therefore, the transcription profiles presented here represent the integration of the transcript levels of different homeologs of each gene. Primer efficiencies were calculated using five 4-fold cDNA dilutions (1:1, 1:4, 1:16, 1:64 and 1:256) in duplicate as well as checking for amplification in a negative control without DNA. The efficiency of the primers used in this study ranged from 86.8% to 99.5% (Additional file 1 table S4). Specificity was checked by analyzing dissociation curves ranging from 60°C to 94°C. Primers for the internal control (*ACTIN*) and conserved primers that amplify all GPC paralogous and homeologous genes in a region outside the RNAi construct have been described previously [3]. The *GPC-6B *copy is deleted in Bobwhite [3].

The RNA equivalent of 1μg cDNA was synthesized using QuantiTect cDNA synthesis kit (QIAGEN) and samples diluted to 10 ng/μl. qRT-PCR reactions included 1 μl cDNA corresponding to 10 ng of total RNA, 10 μl QuantiTect SYBR Green PCR mix (QIAGEN) 0.5 μl of both forward and reverse primers (10 μM, final concentration 250 nM), and 4 μl of water in a 20 μl final reaction volume. qRT-PCR reactions were carried out using an ABI Prism 7000 sequence detection system (Applied Biosystems) using the following cycling conditions: 50°C - 2 min, 95°C - 15 min, 40 cycles of 95°C - 15 sec, 60°C - 1 min. For each validation, 10 biological replicates were used per genotype. The 2^-ΔΔ*C*t ^method [36] was used to normalize and calibrate transcript values relative to the endogenous *ACTIN *control. Within analyses, the same calibrator was used for all genes so the scales of their linearized values are comparable. These linearized values represent the number of RNA copies per copy in the calibrator sample.

## Authors' contributions

DC, SPP, AD, EA, CU, TF and JD conceived and designed the experiments. DC, SPP, AD, MWC, EA carried out the experiments; DC, SPP, and JD performed data analysis; DC and EA performed bioinformatics analyses; DC, SPP, and JD performed the statistical analyses; JD, MWC and AD helped with the interpretation of the results; DC, SPP and JD drafted the manuscript. All authors revised and approved the final version of the manuscript.

## Supplementary Material

Additional file 1 **Figure S1** - WT and GPC-RNAi plants 22 days after anthesis. **Figure S2 **- Frequency distribution of lengths of 454 reads (A) and assembled transcripts (B). **Figure S3 **- Relative distribution of counts coverage of 454 and Illumina data (contigs and singletons). **Figure S4 **- Boxplots showing the distributions of raw (left) and normalized (right) Illumina counts. **Figure S5 **- Sample clustering based on counts of Illumina reads mapped on singletons (left) and unigenes (right). **Figure S6 - **Principal component analysis of the Illumina data. **Figure S7 - **Transcript levels of isogroups validated by qRT-PCR (Table 1*P*≤0.05) and not included in Figure 5 across a senescing time course. **Table S1 **- Summary of 454 sequencing results. **Table S2 **- Summary of Illumina sequencing results. **Table S3 **- Summary of Illumina reads counts. **Table S4 **- Isogroups analysed by qRT-PCR, homeologues, primer sequences and primer efficiencies. **Table S5 - **Percent distribution of the functional grouping of the singletons based on GO-slim annotation. **Table S6 - **Abundance of transposable elements in the assembled transcriptome and in the singletons and their differential expression in WT and GPC-RNAi flag leaves. Click here for file

Additional file 2 **Multi-fasta file with the sequences of 146,671 singletons**. Click here for file

Additional file 3 **Normalized Illumina counts of reads mapped onto 454 contigs with calculated P-values for MWW, edgeR and DESeq statistical analyses**. The table includes also isotigs and contigs IDs with the corresponding GenBank accession number and the associated GOSlim terms. Cell color-coding: red color corresponds to ≥2 fold up-regulation, green color corresponds to ≥ 2 fold down-regulation, yellow color corresponds to *P*≤0.01, and orange color corresponds to *P*≤0.05. Click here for file

Additional file 4 **Normalized Illumina counts of reads mapped onto 454 singletons with calculated *P-*values for MWW, edgeR and DESeq statistical analyses**. Cell color-coding: red color corresponds to ≥2 fold up-regulation, green color corresponds to ≥ 2 fold down-regulation, yellow color corresponds to *P*≤0.01, and orange color corresponds to *P*≤0.05. Click here for file

Additional file 5 **Normalized Illumina counts of reads mapped onto wheat unigenes with the calculated *P-*values for MWW, edgeR and DESeq**. Cell color-coding: red color corresponds to ≥2 fold up-regulation, green color corresponds to ≥ 2 fold down-regulation, yellow color corresponds to *P*≤0.01, and orange color corresponds to *P*≤0.05. Click here for file

Additional file 6 **Set of 815 isogroups differentially regulated at both edgeR and DESeq tests (*P*≤0.01)**. Cell color-coding: red color corresponds to ≥2 fold up-regulation, green color corresponds to ≥ 2 fold down-regulation, yellow color corresponds to *P*≤0.01, and orange color corresponds to *P*≤0.05. Data are sorted by fold-change. Click here for file

Additional file 7 **Set of 691 isogroups differentially regulated at edgeR (*P*≤0.01) DESeq tests (*P*≤0.01) and MWW (*P*≤0.05) tests**. Cell color-coding: red color corresponds to ≥2 fold up-regulation, green color corresponds to ≥ 2 fold down-regulation, yellow color corresponds to *P*≤0.01, and orange color corresponds to *P*≤0.05. Data are sorted by fold-change. Click here for file

Additional file 8 **Set of 748 isogroups similar to transposable elements**. Cell color-coding: red color corresponds to ≥2 fold up-regulation, green color corresponds to ≥ 2 fold down-regulation, yellow color corresponds to *P*≤0.01, and orange color corresponds to *P*≤0.05. Click here for file
