# Supplementary material for: Effect of the down-regulation of the high Grain Protein Content (GPC) genes on the wheat transcriptome during monocarpic senescence
Source: BMC Genomics. 2011 Oct 7;12:492. doi: 10.1186/1471-2164-12-492 (PMC3209470; doi:10.1186/1471-2164-12-492)
Supplement: Additional file 1 — Figure S1 - WT and GPC-RNAi plants 22 days after anthesis. Figure S2 - Frequency distribution of lengths of 454 reads (A) and assembled transcripts (B). Figure S3 - Relative distribution of counts coverage of 454 and Illumina data (contigs and singletons). Figure S4 - Boxplots showing the distributions of raw (left) and normalized (right) Illumina counts. Figure S5 - Sample clustering based on counts of Illumina reads mapped on singletons (left) and unigenes (right). Figure S6 - Principal component analysis of the Illumina data. Figure S7 - Transcript levels of isogroups validated by qRT-PCR (Table 1P≤0.05) and not included in Figure 5 across a senescing time course. Table S1 - Summary of 454 sequencing results. Table S2 - Summary of Illumina sequencing results. Table S3 - Summary of Illumina reads counts. Table S4 - Isogroups analysed by qRT-PCR, homeologues, primer sequences and primer efficiencies. Table S5 - Percent distribution of the functional grouping of the singletons based on GO-slim annotation. Table S6 - Abundance of transposable elements in the assembled transcriptome and in the singletons and their differential expression in WT and GPC-RNAi flag leaves. [file 1471-2164-12-492-S1.PDF]

Additional File S1

Figures

Figure S1 - WT and GPC-RNAi plants 22 days after anthesis.

Figure S2 - Frequency distribution of lengths of 454 reads (A) and assembled transcripts (B).

Figure S3 – Relative distribution of counts coverage of 454 and Illumina data (contigs and singletons).

Figure S4 - Boxplots showing the distributions of raw (left) and normalized (right) Illumina counts.

Figure S5 - Sample clustering based on counts of Illumina reads mapped on singletons (left) and unigenes (right).

Figure S6 - Principal component analysis of the Illumina data.

Figure S7 - Transcript levels of isogroups validated by qRT-PCR (Table 1, $P \leq 0.05$) and not included in Figure 5 across a senescing time course.

Tables

Table S1 - Summary of 454 sequencing results

Table S2 - Summary of Illumina sequencing results

Table S3 - Summary of Illumina reads counts

Table S4 – Isogroups analysed by qRT-PCR, homologues, primer sequences and primer efficiencies.

Table S5 - Percent distribution of the functional grouping of the singletons based on GO-slim annotation

Table S6 - Abundance of transposable elements in the assembled transcriptome and in the singletons and their differential expression in WT and GPC-RNAi flag leaves.

FIGURES

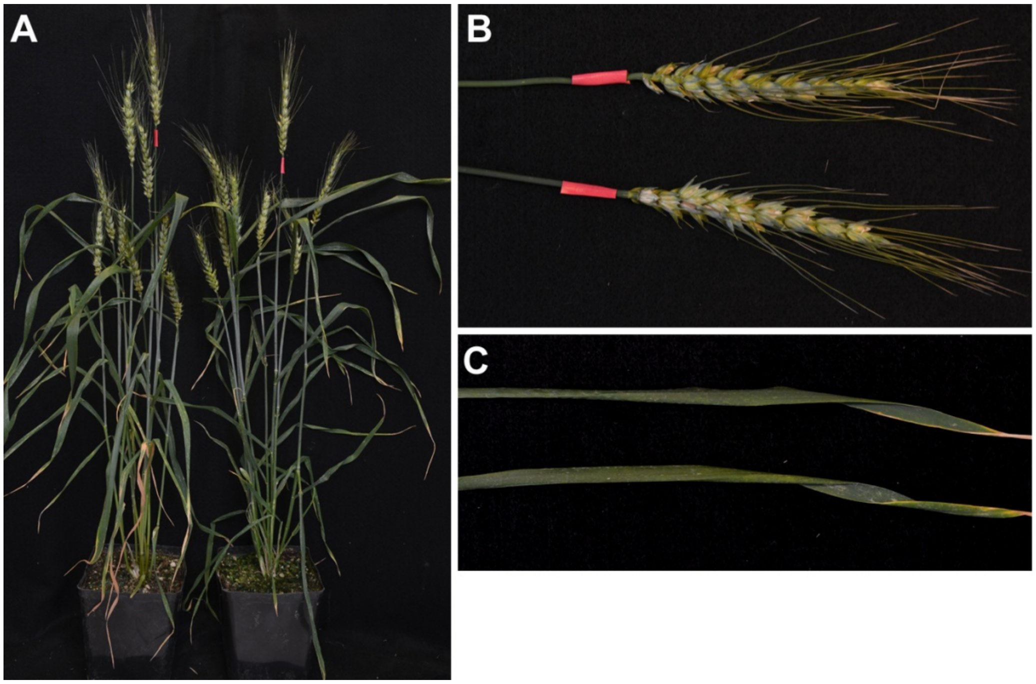

Figure S1 – WT and GPC-RNAi plants 22 days after anthesis.

(A) No visual symptoms of senescence are evident at 22 DAA in either WT (left) or GPC-RNAi plants (right) and there are no visible phenotypic differences between the two genotypes.

(B&C) Close-up images of the ears (B) and flag leaves (C) from WT (top) and GPC-RNAi plants (bottom).

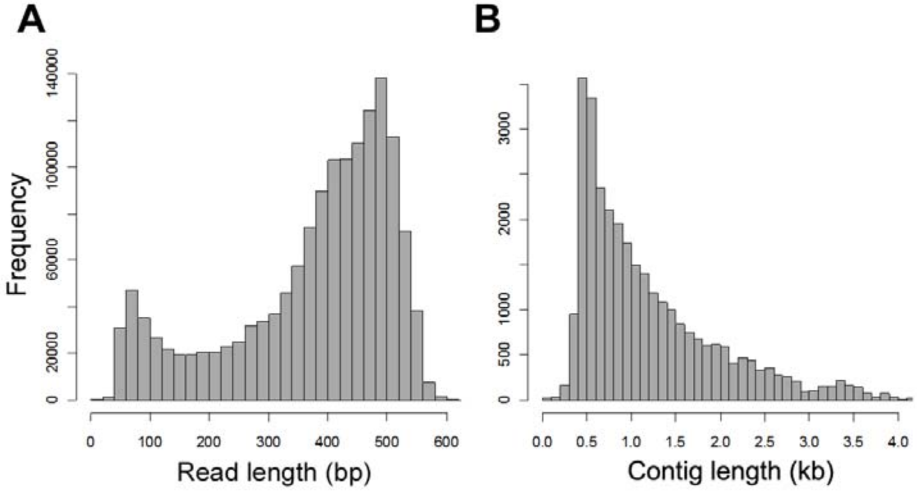

Figure S2 – Frequency distribution of lengths of 454 reads (A) and assembled transcripts (B).

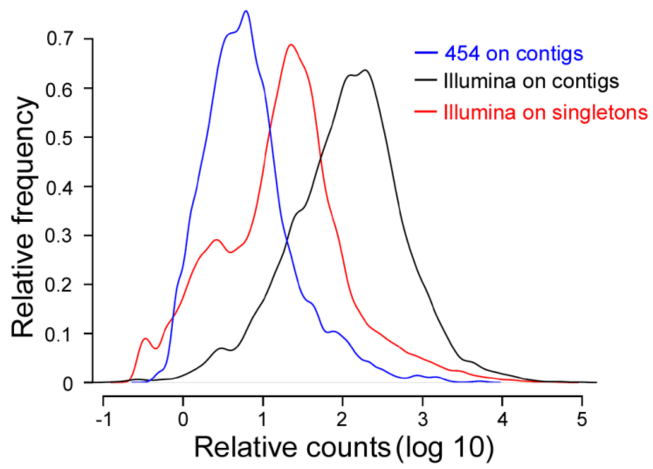

Figure S3 – Relative distribution of counts coverage of 454 and Illumina data.

Relative counts are calculated as the average of counts across the WT and GPC RNAi libraries divided by the length (in Kbp) of the contig or the singleton where the reads map.

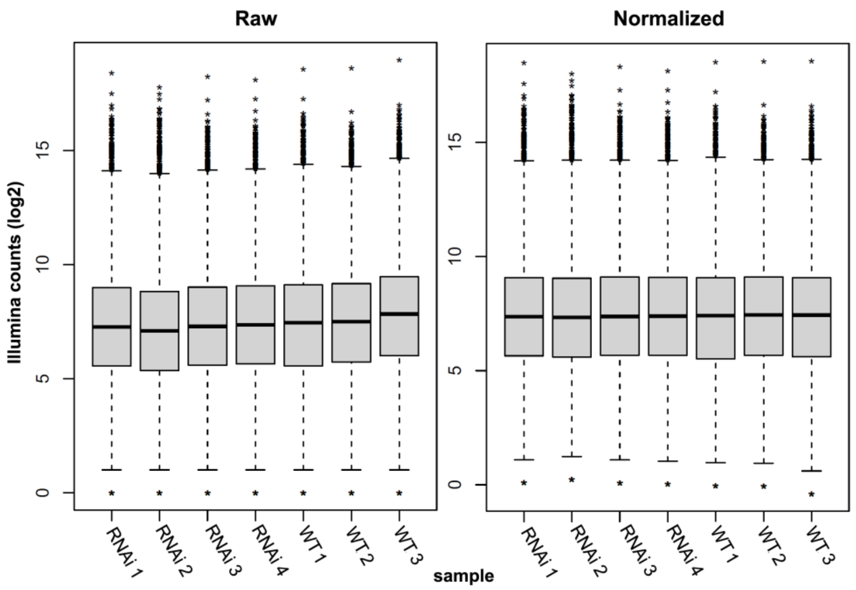

Figure S4 – Boxplots showing the distribution of raw (left) and normalized (right) Illumina counts.

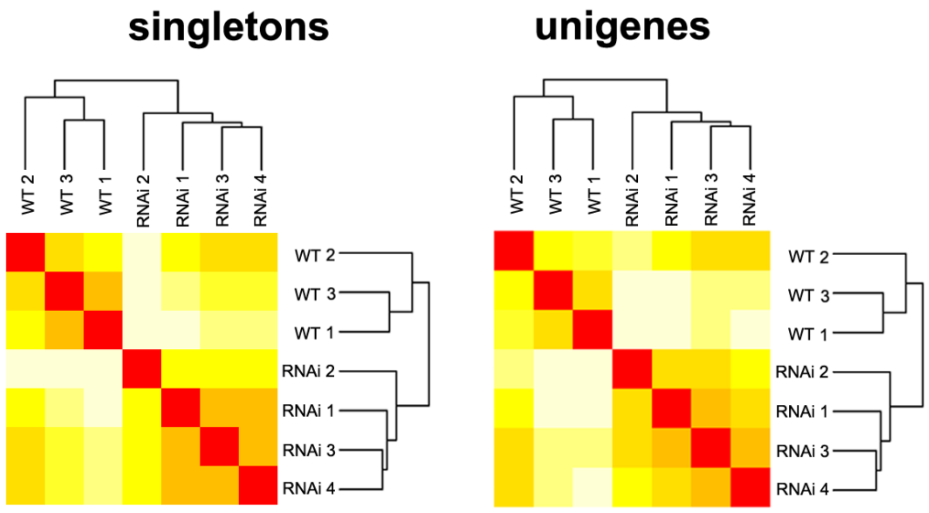

Figure S5 - Sample clustering based on counts of Illumina reads mapped on singletons (left) and unigenes (right).

Dendrogram represents the hierarchical clustering of samples as determined by Euclidean distance. The heat map shows a false color representation of the Euclidean distance matrix (from red for zero distance to white for large distance).

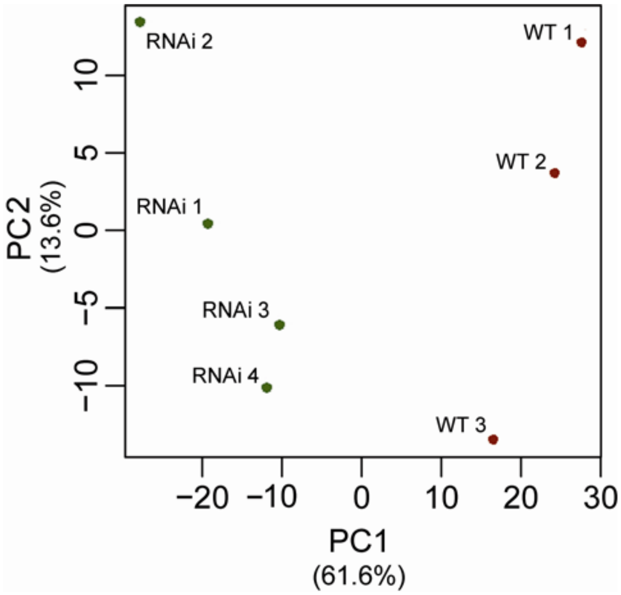

Figure S6 – Principal component analysis of the Illumina data.

Biological replicates from the two genotypes (green=GPC RNAi, red=WT) are distinguished by PC1, which accounts for most of the variation (61.6%).

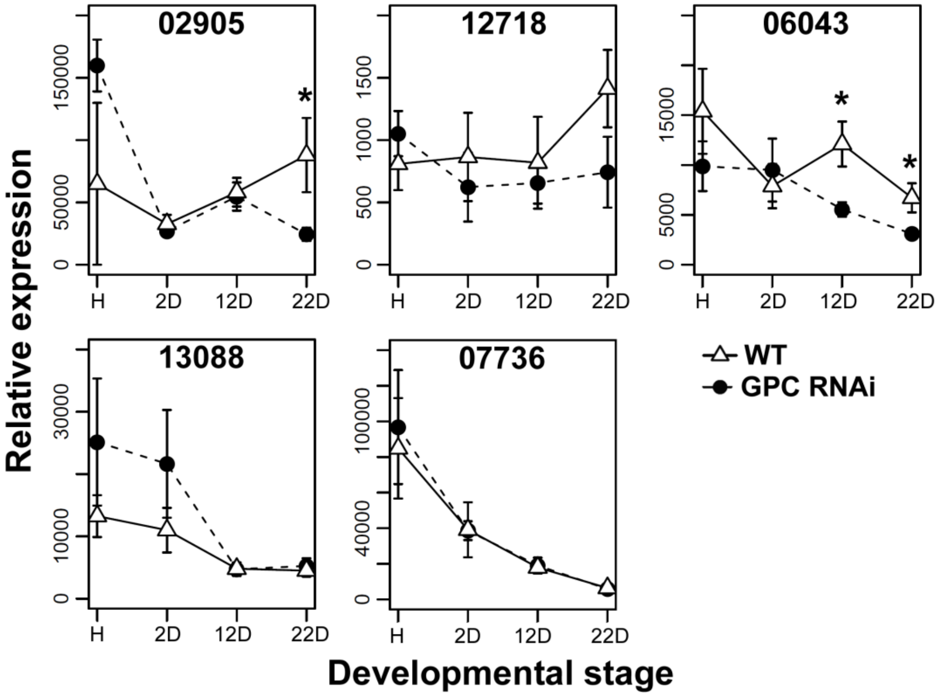

Figure S7 - Transcript levels of selected isogroups across a senescing time course. Expression levels in WT and GPC-RNAi plants were determined using qRT-PCR at four points across a senescing time course. (H = heading, D = days after anthesis). Transcript levels presented as normalized linearized values using the $2(^{-\Delta\Delta Ct})$ method [34], where Ct is the threshold cycle. Values are corrected using the same calibrator meaning gene expression is comparable between isogroups.

TABLES

Table S1 - Summary of 454 sequencing results

	Reads	
**Sample**	**Number**	**Median Length**
**WT rep 1**	206,856	419
**WT rep 2**	186,633	430
**WT rep 3**	161,296	403
**WT rep 4**	176,471	404
**GPC RNAi rep 1**	223,363	420
**GPC RNAi rep 2**	195,811	420
**GPC RNAi rep 3**	162,352	416
**GPC RNAi rep 4**	157,035	401

Table S2 - Summary of Illumina sequencing results

	Raw Reads		Trimmed and filtered reads	
**Sample**	**Number**	**Length**	**Number**	**Median Length**
**WT rep 1**	31,217,550	85	20,835,506	59
**WT rep 2**	30,476,425	85	20,295,510	47
**WT rep 3**	40,409,126	85	25,020,382	46
**WT rep 4***	33,589,557	85	21,059,385	43
**GPC RNAi rep 1**	31,959,282	85	20,000,920	43
**GPC RNAi rep 2**	23,547,242	85	19,958,148	59
**GPC RNAi rep 3**	25,892,123	85	19,562,481	47
**GPC RNAi rep 4**	25,925,925	85	19,632,629	46

* Sample eliminated from statistical analyses due to library contamination

Table S3 - Summary of Illumina read counts

Sample	Total counts	%[1]	Reference seq hit	%[2]	Coverage[3] [nt/nt]	Counts / contig Average	Counts / contig Median
**WT rep 1**	12,894,847	61.9	30,069	98.6	26.9	429	120
**WT rep 2**	12,163,985	59.9	30,158	98.9	20.2	403	124
**WT rep 3**	15,786,809	63.1	30,153	98.9	25.6	524	155
**WT rep 4***	13,275,642	63.0	30,118	98.8	20.2	441	108
**GPC RNAi rep 1**	12,375,313	61.9	30,123	98.8	18.8	410	110
**GPC RNAi rep 2**	12,051,198	60.4	30,005	98.4	25.2	402	101
**GPC RNAi rep 3**	11,706,878	59.8	30,129	98.8	19.4	389	111
**GPC RNAi rep 4**	11,857,265	60.4	30,142	98.8	19.3	393	118

* Sample eliminated from statistical analyses due to library contamination

[1] Percentage of the Illumina reads mapping to the consensus (Total counts / Total reads).

[2] Percentage of the 454 consensus sequences matched at least once by Illumina reads.

[3] Coverage is calculated as number of nucleotides of Illumina reads per nucleotide of 454 reference [(number of matching reads x median length of reads) / (number of reference sequence hit x median 454 sequence length)].

Table S4. Isogroups analyzed by qRT-PCR. Isogroup number, closest homolog with either rice locus, GenBank or SwissProt identifier, number of isotigs within each contig and primers used in the qRT-PCR experiments with their respective efficiencies.

Isogroup	Rice homolog	Rice annotation	Isotigs	Forward Primer (5' – 3')	Reverse Primer (5' – 3')	Efficiency (%)
01211	LOC_Os05g50340.1	MYB family transcription factor	3[a]	AATGGCCAAGGGATGATACA	GACACCATGCACACGCTTAT	99.48
02905	BAK01295	Predicted protein	2[a]	ACAAACCCAGTCATGCCAAA	ACGGCAGAGATGTTTACACG	99.44
03083	BAJ98715	Predicted protein	2[b]	TTGATGCCAAATACCCAACA	GGATGCTGCAAAGGAAAAAGA	94.52
03470	P13194	Photosystem 1 reaction subunit IV	1	CTTTTTCTCGCGTTGGTCTC	TCCTCCTCTCCCTCCTCCTA	92.39
05843	LOC_Os10g28610.1	KIP1	1	GCAGTACCGTTCTCGTCCTC	TGACATGGCTGAGAAAAACG	93.00
06043	LOC_Os04g34490.1	Nodulin	1	TTCCCCACAGGACCAAGTAG	CGCTAATCCAGATCGTCACC	93.87
06482	LOC_Os01g22630.2	ECT	1	ATTTTCCGGCTTTCCAGACT	GGGTAAGGGCTGTAGGAAGG	95.55
06574	LOC_Os01g17430.1	coatomer subunit beta-1	1	TTTCCTCATGCTCTGCCTCT	GACCTTGCGGATGAGGTCTA	97.68
07736	LOC_Os04g53310.1	soluble starch synthase 3 chloroplast precursor	1	GGAATCCGAGGAGATCATCA	TTCGTGGAGGTCCAAGATTC	95.44
07898	LOC_Os05g05470.1	T-complex	1	CATCGGGGAGCTTATGAAGA	GCTTTCTTGGCAACCTCAAAG	97.70
08662	LOC_Os12g14440.1	Jacalin-like lectin domain containing	1	GCTCCTCCAGGGACCTCTAC	TGTAGGAGAAGCCGAAGACG	99.10
10053	LOC_Os08g20200.2	male sterility	1	TACGCCATCCTTTGGAAGTC	GAACACGCTCACCATTCCTT	94.03
10136	Q09134	ABA and environmental stress-inducible protein	1	TTGGAGTCCGTTCATGTTGA	CGCCCGTTACAGTATTGGAT	92.75
10620	LOC_Os03g31300.1	chaperone clpB 1	1	GACCCTGAGGGCAAGTATGA	TGCACCTACGGATTTCATCA	93.12
10811	LOC_Os05g12320.1	nodulin MtN3 family putative	1	AATGTATGGCTCTCCGTTGG	GCCCATACAGCATCCAGAGT	94.30
10940	LOC_Os08g44820.3	no apical meristem	1	GCGACTCCCAGTGGTACTTC	TCTTCTTCATCCCGACAACC	92.29
11278	LOC_Os07g35310.2	TKL IRAK DUF26-lc.12	1	GTGCACGTTGGAGTAGGTGA	GACTACAAGGACGCCACCAT	88.14
12718	BAJ93182	Protein kinase	1	TAGCAGTGATGCCTGTTTGG	CATGCTCGTCAGAGCTGGTA	98.27
13088	LOC_Os01g41750.1	expressed protein	1	GCCTTGAAGGACAGAAGTGC	CCGTCTGATTCCCTTGCTAA	95.68
13287	LOC_Os03g11900.1	transporter family	1	GACCACCATAGGCGTGATCT	GAGAAAATCAGCCCACCTGA	87.25
13722	LOC_Os01g06790.1	disease resistance	1	CCTCACATGCTACTGCTCCA	ATGACGAGAGGTTGGGTTTG	97.40
14133	P93298	ATP synthase subunit a-1	1	CAAATGGGACTCCAATACCC	AAGAGTAAACGGTCGAATTGATG	96.13
14210	LOC_Os04g35790.3	GLTP domain containing	1	AAGATCGCGTTGAAGCTCAT	ATGGATGTCGTCGAGAAAGG	86.88
14599	LOC_Os10g02720.1	OsWAK99 - OsWAK receptor-like kinase	1	GCGCATGCTAGTTGATTCCT	TGATCGTGGTGCTCCAGTAA	90.23

a = Alternative splicing variants, b = Homoeologous isotigs,

Table S5 - Percent distribution of the functional grouping of the singletons based on GO-slim annotation

Singletons

**Accession**	**Ontology**	**Total** [a]	**Up-regulated** [a]	**Down-regulated** [b]
GO:0019538	protein metabolic process	4.4	7.6	0
GO:0009987	cellular process	5.7	4.4	8.8
GO:0006950	response to stress	18.2	27.9	11.2
GO:0006810	transport	2.2	5.5	1.5
GO:0009719	response to endogenous stimulus	6.5	2.9	6.7
GO:0009056	catabolic process	3.5	2.4	1.5
GO:0009607f	response to biotic stimulus	3.8	4.4	3.1
GO:0007165	signal transduction	7.7	1.5	7.2
GO:0006464	protein modification process	10	0	4.3
GO:0006350	transcription	1.3	1.5	1.9
GO:0009628	response to abiotic stimulus	7.7	26.5	6.5
GO:0016043	cellular component organization and biogenesis	2.5	0	5.6
GO:0008152	metabolic process	4.5	17.6	4.4
GO:0009058	biosynthetic process	2.3	1.5	3.1
GO:0006412	translation	2.2	0	3.4
	Others	17.5	8.8	18.3

Unigenes

**Accession**	**Ontology**	**Total** [a]	**Up-regulated** [a]	**Down-regulated** [a]
GO:0019538	protein metabolic process	4.7	3.5	7.9
GO:0009987	cellular process	7.7	7.0	13
GO:0006950	response to stress	12.3	8.5	10
GO:0006810	transport	4.2	6.3	5.3
GO:0009719	response to endogenous stimulus	6.5	5.2	5.2
GO:0009056	catabolic process	4.1	6.3	3.3
GO:0009607	response to biotic stimulus	3.5	4.9	4.2
GO:0007165	signal transduction	6.3	5.4	5.5
GO:0006464	protein modification process	7.9	8.0	3.3
GO:0006350	transcription	5.2	5.6	2
GO:0009628	response to abiotic stimulus	4.9	3.1	6.4
GO:0016043	cellular component organization and biogenesis	3.7	3.5	2.4
GO:0008152	metabolic process	2.8	5.2	2.7
GO:0009058	biosynthetic process	3.1	3.8	5.2
GO:0006412	translation	5.2	5.6	2
	Others	17.9	18.1	21.6

[a] Significantly different between genotypes ($P \leq 0.01$ edgeR and DEseq).

Table S6 - Abundance of transposable elements in the assembled transcriptome and in the singletons and their differential expression in WT and GPC-RNAi flag leaves.

	Isogroups						Singletons					
**Superfamily**	**Total**		**UP** [C]		**DOWN** [C]		**Total**		**UP** [C]		**DOWN** [C]	
CACTA	101	(14)[a]	10	(25)	0	(0)	465	(12)	1	(4)	1	(50)
Copia	91	(12)	5	(13)	1	(17)	610	(15)	1	(4)	0	(0)
Gypsy	150	(20)	24	(60)	0	(0)	1407	(35)	11	(41)	1	(50)
Harbinger	17	(2)	1	(3)	0	(0)	46	(1)	0	(0)	0	(0)
HAT	2	(0)	0	(0)	0	(0)	13	(0)	0	(0)	0	(0)
Helitron	4	(1)	0	(0)	1	(17)	19	(0)	0	(0)	0	(0)
LINE	10	(1)	0	(0)	0	(0)	50	(1)	0	(0)	0	(0)
Mariner	273	(36)	4	(0)	3	(50)	845	(21)	14	(52)	0	(0)
Mutator	5	(1)	0	(0)	0	(0)	64	(2)	0	(0)	0	(0)
unknown	95	(13)	0	(0)	1	(17)	491	(12)	0	(0)	0	(0)
Total	748		44	(87)[b]	6	(13)[b]	4010		27	(93)[b]	2	(7)[b]

[a] Values in parentheses are percentages over the sum of values per column.

[b] Values in parentheses are percentages over the total number of up- or down-regulated elements.

[c] Differential regulation is based on both edgeR and DESeq test at $P \leq 0.01$.
